# Supplementary material for: Synthesis and Micellization Behavior of Amphiphilic Block Copolymers of Poly(N-vinyl Pyrrolidone) and Poly(Benzyl Methacrylate): Block versus Statistical Copolymers
Source: Polymers (Basel). 2023 May 8;15(9):2225. doi: 10.3390/polym15092225 (PMC10181249; doi:10.3390/polym15092225)
Supplement: Supplementary file 1 [file polymers-15-02225-s001.zip › polymers-2361307-supplementary.pdf]

Supplementary Material: Synthesis and Micellization Behavior of Amphiphilic Block Copolymers of Poly(N-vinyl Pyrrolidone) and Poly(Benzyl Methacrylate): Block versus Statistical Copolymers

Nikoletta Roka and Marinos Pitsikalis

Supporting Information Section

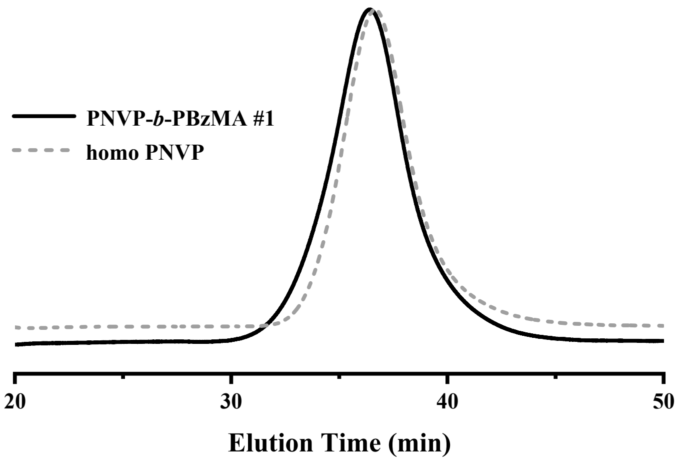

Figure S1: SEC traces from the synthesis of the block copolymer PNVP-*b*-PBzMA#1

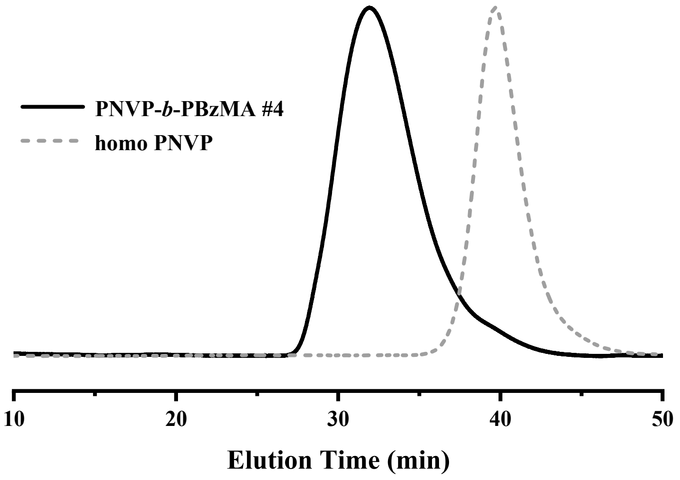

Figure S2: SEC traces from the synthesis of the block copolymer PNVP-*b*-PBzMA#4

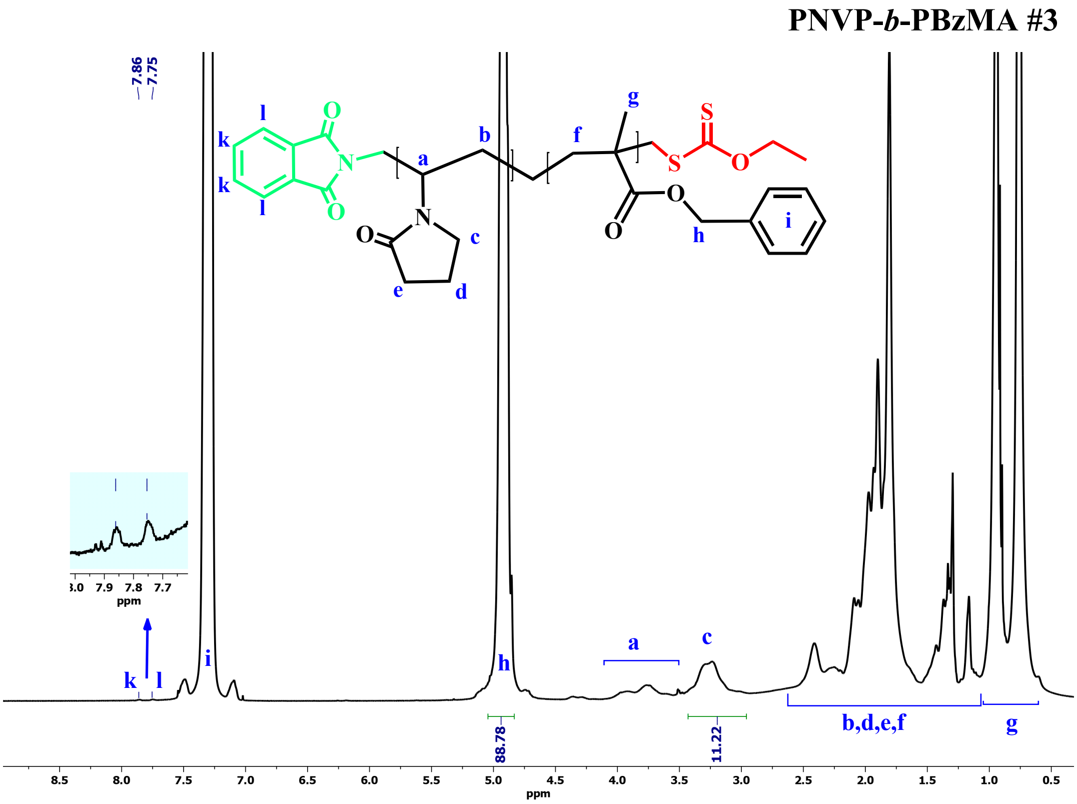

Figure S3. 400 MHz ^{1}H NMR spectrum of the block copolymer PIBMA-*b*-PNVP#3

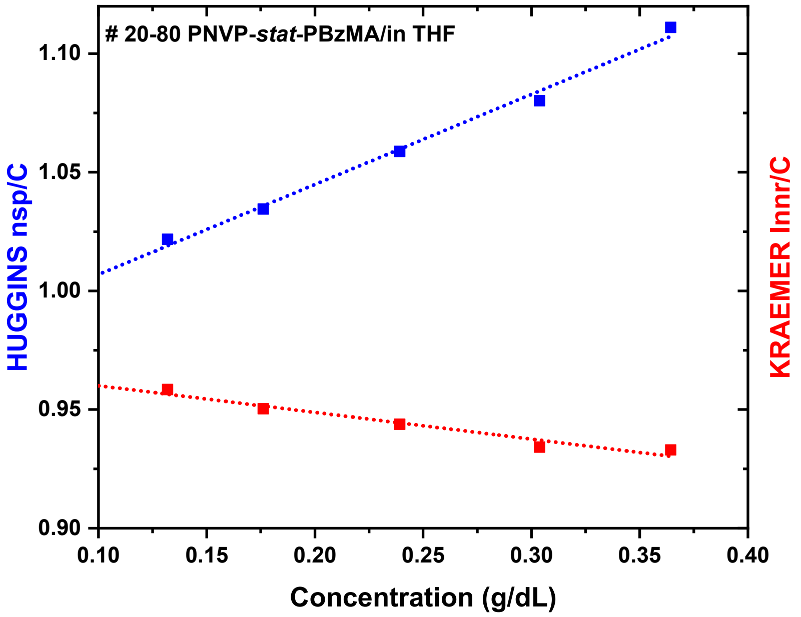

Figure S4. Dilute solution viscometry plots of the sample 20/80

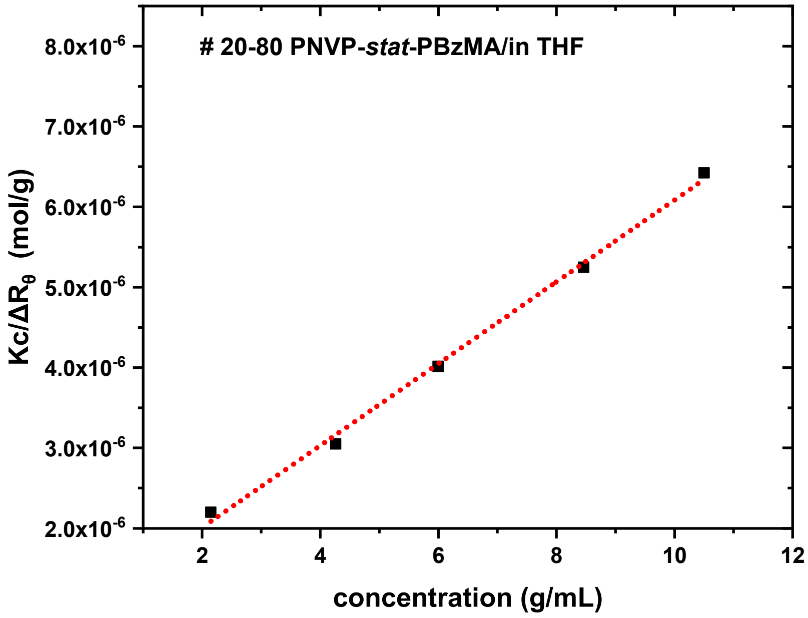

Figure S5. SLS plots of the sample 20-80

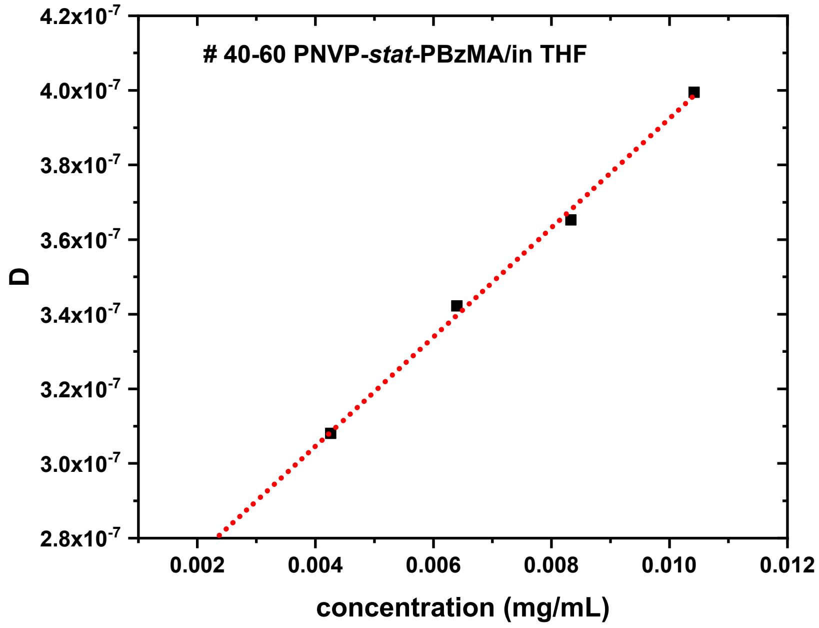

Figure S6. DLS plots of the sample 40-60

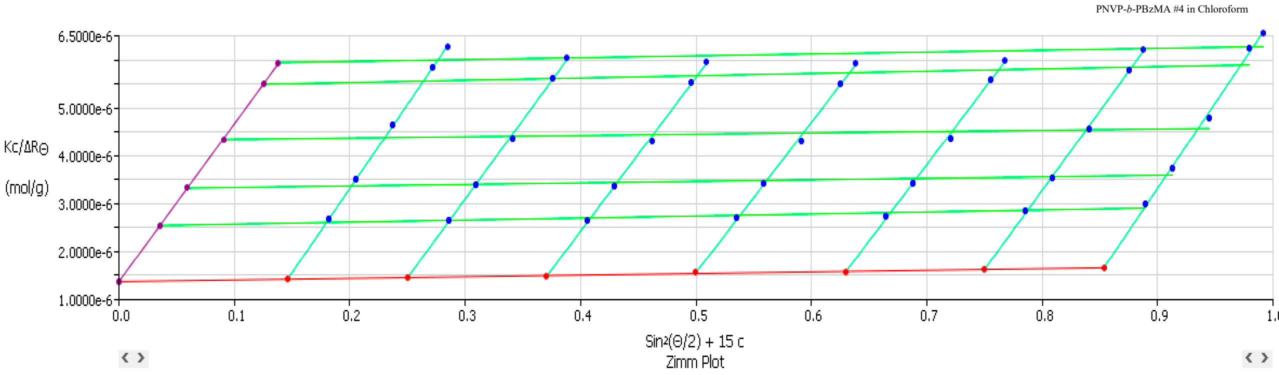

Figure S7. SLS plots of the sample PNVP-*b*-PBzMA#4 in $CHCl_3$.

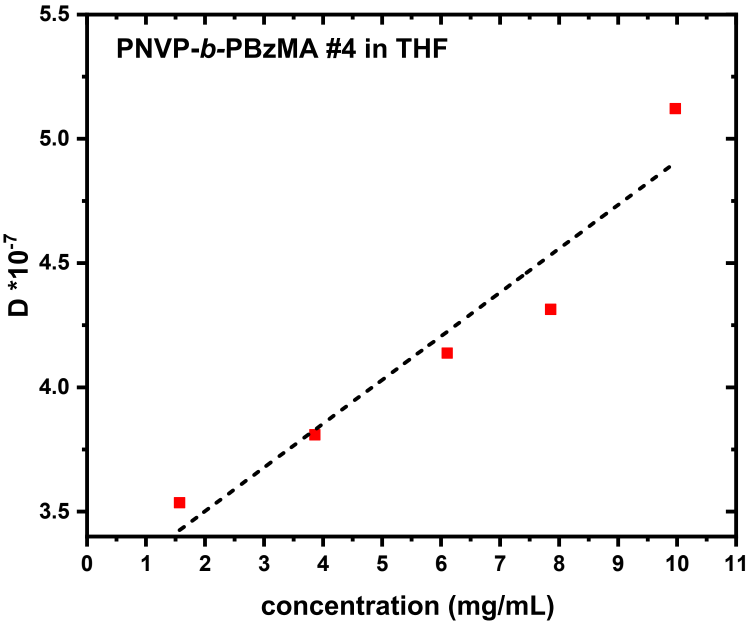

Figure S8. DLS plots of the sample PNVP-*b*-PBzMA#4 in THF

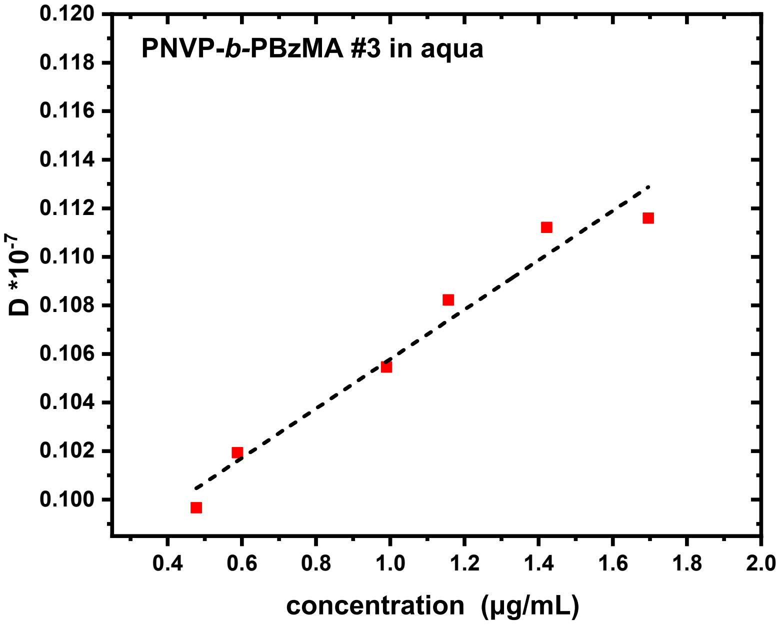

Figure S9. DLS plots of the sample PNVP-*b*-PBzMA#3 in aqueous solution

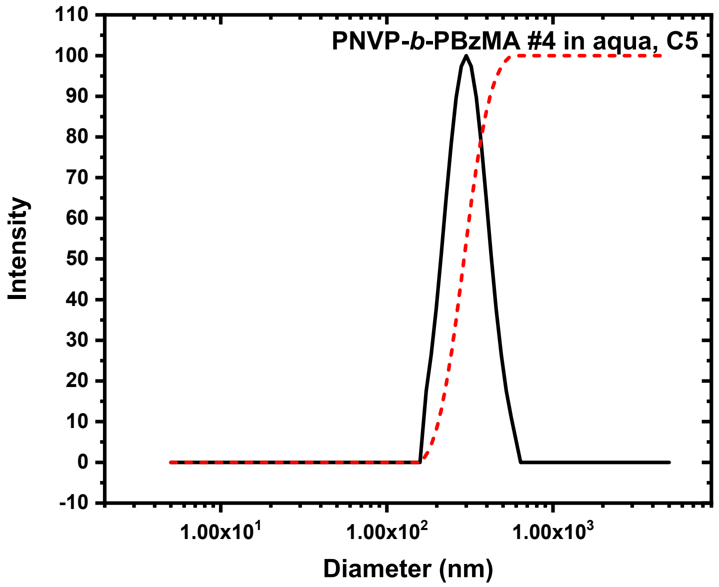

Figure S10. CONTIN plot for the sample PNVP-*b*-PBzMA#4 in aqueous solution

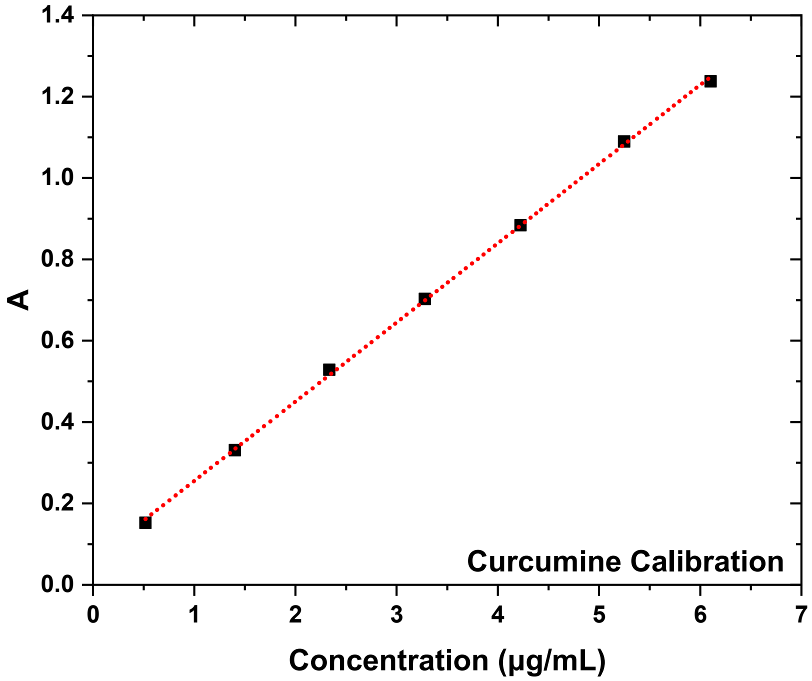

Figure S11. Calibration curve for curcumin from UV-Vis spectroscopy measurements at 423.50 nm
